# Taxonomy and phylogeny of *Dacrymyces* (Dacrymycetales, Basidiomycota) with descriptions of a new species from China

**DOI:** 10.3389/fmicb.2026.1875564

**Published:** 2026-08-06

**Authors:** Zhan-Bo Liu, Ke-Ni Meng, Jian-Ling Zhang, Ying-Ge Fan, Han Liu, Guang-Peng Weng, Xiao-Bin Liu, Jing-Xuan Xu, Yu-Tong Zhang, Qing-Chao Zeng, Zhou-Jie Du, Han Zhao, Shu-Jiang Li

**Affiliations:** 1 College of Forestry, Sichuan Agricultural University, Chengdu, China; 2 Forest Ecology and Conservation in the Upper Reaches of the Yangtze River Key Laboratory of Sichuan Province, Chengdu, China; 3 Sichuan Mt. Emei Forest Ecosystem National Observation and Research Station, Leshan, China; 4 Ganzi Institute of Forestry Research, Kangding, China; 5 Gateway Antarctica, Centre for Antarctic Studies and Research, School of Earth & Environment, University of Canterbury, Christchurch, New Zealand; 6 Department of Plant Pathology, College of Plant Protection, China Agricultural University, Beijing, China; 7 Songpan Forestry and Grassland Bureau, Aba, China; 8 Bamboo Resource Conservation and Utilization Key Laboratory of Sichuan Province, Leshan, China

**Keywords:** brown-rot fungi, classification, Dacrymycetaceae, jelly fungi, molecular phylogeny

## Abstract

A new jelly fungus, *Dacrymyces abaensis* sp. nov., is described from the subalpine coniferous forests of Sichuan, China, based on morphology and ITS+nLSU phylogeny. The species grows on rotten wood of *Picea* and forms a distinct lineage within *Dacrymyces*. It is characterized by sessile, cerebriform, orange-yellow gelatinous basidiomata; subclavate probasidia (109–120 × 5.5–9 μm) that become bifurcate at maturity; clavate, thin-walled hyphidia (39–61 × 3.5–5.5 μm); and large, navicular basidiospores (36.6–56 × 11.9–15.6 μm) with up to 15 transverse septa and several longitudinal septa, germinating via conidia and germ tubes. A detailed description, illustrations, and the results of the phylogenetic analysis for the new species are provided. In addition, the new species is compared with closely related taxa.

## Introduction

Fungi play indispensable roles in ecosystems, functioning as vital decomposers, pathogens, and symbiotic partners for diverse plant and animal species (Tedersoo et al., 2014; James et al., 2020; Wu et al., 2022; Yuan et al., 2023; Hyde et al., 2024; Nie et al., 2025; Wijayawardene et al., 2025; Zhao et al., 2025, 2026). Some fungi have significant economic value as sources of food and medicine (Dai and Yang, 2008; Wu et al., 2019, 2022, 2026). In forest habitats, fungi are the main regulators of soil carbon cycling. In addition, they enhance mineral absorption in plants and alleviate stress caused by carbon limitations (Wei and Dai, 2004; Rokas et al., 2018; Wu et al., 2022).

The genus *Dacrymyces* Nees (Dacrymycetaceae, Dacrymycetales) represents an ecologically and taxonomically significant group of wood-decaying fungi (Wu et al., 2026). Species of this genus typically inhabit dead trees, stumps, and fallen trunks or branches of both angiosperms and gymnosperms, playing a vital role in wood decomposition by causing brown rot (Shirouzu et al., 2009; Zamora and Ekman, 2020). Morphologically, *Dacrymyces* exhibits extreme phenotypic plasticity. The basidiomata are soft- to firm-gelatinous, range in color from cream to orange-yellow, and vary greatly in shape, initially developing as pustulate structures and later transforming into pulvinate, discoid, cupulate, cerebriform, or spathulate to flabellate. Microscopically, the genus is characterized by bifurcate (Y-shaped) basidia and basidiospores that are usually transversely septate and occasionally develop longitudinal septa at maturity (McNabb, 1973; Shirouzu et al., 2017; Lian et al., 2022; Mendes-Alvarenga and Gibertoni, 2022; Ma et al., 2024; Wang and Bau, 2024).

Historically, the taxonomic circumscription of *Dacrymyces* has undergone significant expansion, leading to considerable complexity. The genus was originally established by Nees von Esenbeck (1816) to accommodate a single type species, *D. stillatus* Nees, which was narrowly characterized by sessile, pustulate to pulvinate basidiomata and hyphae lacking clamp connections. Over time, however, the morphological boundaries were broadened to include species with stipitate, morphologically diverse basidiomata, and hyphae possessing clamp connections (Liu and Fan, 1989, 1990). Consequently, *Dacrymyces* has become a massive repository for dacrymycetous fungi, with 233 specific and infraspecific names currently recorded in Index Fungorum (accessed 17 April 2026); of these, 154 names are currently accepted as legitimate species.

Despite this extensive morphological documentation, the taxonomy and phylogeny of *Dacrymyces* remain highly ambiguous. The extreme phenotypic variability means that no single morphological synapomorphy can reliably distinguish *Dacrymyces* from related genera (Shirouzu et al., 2007, 2009, 2017). The advent of molecular phylogenetics has further exposed these taxonomic challenges. Early analyses based on nLSU sequences (Weiß and Oberwinkler, 2001; Weiss et al., 2004; Shirouzu et al., 2007) revealed that *Dacrymyces* is highly polyphyletic. Subsequent multigene phylogenetic studies have repeatedly corroborated this deeply polyphyletic topology, showing that *Dacrymyces* species are widely scattered across various branches of Dacrymycetaceae (Shirouzu et al., 2013, 2017; Zamora and Ekman, 2020).

During investigations into the diversity of wood-rotting fungi, two gelatinous specimens were collected in Sichuan Province, China. Their morphology corresponds to the concept of *Dacrymyces*. To confirm their affinity, phylogenetic analyses based on ITS+nLSU sequences were performed. Both morphological characteristics and molecular evidence demonstrate that these two gelatinous specimens represent a new species of *Dacrymyces*.

## Materials and methods

### Collection

The fungal materials were collected from Sichuan Province, China. For each specimen, we recorded the specific habitat, collection date, and exact locality and deposited the voucher specimens in the Fungarium of Sichuan Agricultural University (SAUFC, China). Notably, the holotype was discovered collected in from the Mounigou Scenic Area, Songpan County, Aba, Sichuan Province, on rotten wood of *Picea* (103° 31′E, 32° 37’N, altitude 3,002 m). The vegetation type is a subalpine coniferous forest. The detailed information for the specimens examined is as follows: 31 Aug 2025, Z. B. Liu 1105 (holotype SAUFC 001105), Z. B. Liu 90 (paratype SAUFC 000090).

### Morphological studies

Macro-morphological descriptions are based on field notes and observations of dry herbarium specimens. Microscopic structures were photographed using a Nikon Digital Sight DS-L3 (Japan) or Leica ICC50 HD (Japan) camera. Microscopic measurements were obtained from slide preparations of dry tissues stained with 1% Congo red solution (Ma et al., 2024). The following abbreviations are used: KOH = 5% potassium hydroxide, L = mean spore length (arithmetic average of all spores), W = mean spore width (arithmetic average of all spores), Q = variation in the L/W ratios between the specimens studied, and n (a/b) = number of spores (a) measured from a given number of specimens (b). In presenting spore size variation, 5% of measurements at each end of the range were excluded, and these values are given in parentheses. Special color terms follow Petersen (1996). Herbarium abbreviations follow Thiers (2018). The studied specimens have been deposited in the Fungarium of Sichuan Agriculture University (SAUFC, China).

### DNA extraction, PCR amplification, and sequencing

Total genomic DNA was extracted from dried specimens using a CTAB rapid plant genome extraction kit (Aidlab Biotechnologies Company, Limited, Beijing, China) according to the manufacturer’s instructions, with some modifications (Dong et al., 2024; Yang et al., 2025). The ITS regions are amplified with primers ITS4 and ITS5 (White et al., 1990). The nLSU regions were amplified using primers LR0R and LR7 (Vilgalys and Hester, 1990).

The polymerase chain reaction (PCR) procedure for ITS is was as follows: initial denaturation at 95 °C for 3 min, followed by 35 cycles at 94 °C for 40 s, 58 °C for 45 s, and 72 °C for 1 min, with a final extension of at 72 °C for 10 min (Dong et al., 2025). The PCR procedure for nLSU was as follows: Initial denaturation at 94 °C for 1 min, followed by 35 cycles at 94 °C for 30 s, 48 °C for 1 min, and 72 °C for 1.5 min, with a final extension of at 72 °C for 10 min (Deng et al., 2026). The purification and sequencing of the PCR products were conducted by Sangon Biotech (Shanghai) Co., Ltd., Chengdu, China, with the same primers used in the PCR reactions. Species were identified by sequence comparison with accessions in the NCBI database accessions using the BLAST program.

### Phylogenetic analyses

Phylogenetic trees were constructed using ITS+nLSU rDNA sequences, and phylogenetic analyses were performed using maximum likelihood (ML) and Bayesian inference (BI) methods. Sequences of the species and strains were primarily adopted from the ITS+nLSU tree topology, as described by Hongsanan et al. (2025). New sequences generated in this study, along with reference sequences retrieved from GenBank (Table 1), were aligned using MAFFT 7 (Katoh et al., 2019)^1^ with the “G-INS-i” strategy and manually adjusted in BioEdit (Hall, 1999). Unreliably aligned sections were removed before analyses, and we attempted to manually inspect and improve the alignment. The data matrix was edited using the Mesquite v3.81 software (Maddison and Maddison, 2023). The sequence alignment has been deposited in Figshare (10.6084 m9.figshare.32588508). Sequences of *Cerinomyces aculeatus* N. Maek obtained from GenBank were used as outgroups to root the trees in the ITS+nLSU analyses.

**Table 1:** Information on taxa used in phylogenetic analyses.

Species	Sample no.	GenBank accession numbers		Country
		ITS	LSU	
*Calocera bambusicola*	Wu9910 12	FJ195751	—	China
*Calocera cornea*	CBS124 84	AB712437	AB472738	Japan
*Calocera furcata*	H: Spirin 10,949	MW191975	MW159088	Russia
*Calocera furcata*	TU135016	MW191958	MW159087	Estonia
*Calocera fusca*	PDD 107930	LC131405	LC131364	New Zealand
*Calocera glossoides*	CWU6247	MW191968	MW159084	Ukraine
*Calocera guepinioides*	PDD 107969	LC131410	LC131369	New Zealand
*Calocera lutea*	PDD 107841	LC131413	LC131372	New Zealand
*Calocera multiramosa*	HKAS 133171*	PP550874	PP550021	China
*Calocera multiramosa*	HKAS 133172	PP550875	PP550022	China
*Calocera pedicellata*	PDD 107830	LC131415	LC131374	New Zealand
*Calocera ramaria*	CLZhao 31166*	PP399147	PP862915	China
*Calocera sinensis*	Dai22645	OL518939	OL518948	China
*Calocera tibetica*	Dai20171*	MW549777	MW750403	China
*Calocera tibetica*	Dai20178	MW549778	MW750404	China
*Calocera velutina*	FJAU68950*	PP776476	PP776564	China
*Calocera velutina*	FJAU68951	PP776543	PP776565	China
*Calocera viscosa*	AFTOL-ID 1679	DQ520102	DQ520102	Germany
*Cerinomyces aculeatus*	TU135069	MW191883	MW159044	Russia
*Cerinomyces aculeatus*	TU135070	MW191884	MW159045	Russia
***Dacrymyces abaensis***	Liu 1105*	**PZ356994**	**PZ356995**	**China**
***Dacrymyces abaensis***	**Liu 90**	**PZ356993**	**PZ356996**	**China**
*Dacrymyces adpressus*	TUFC12845	AB712447	AB472707	Japan
*Dacrymyces ancyleus*	MAFF241177	AB712448	AB472713	Japan
*Dacrymyces burdsallii*	CFMR: HHB-6908*	AB712444	AB712424	USA
*Dacrymyces capitatus*	Dai 20,023	OL587808	OL546776	China
*Dacrymyces capitatus*	CBS 293.82	AB712450	AB472741	Canada
*Dacrymyces ceraceus*	CFMR: HHB-8969*	AB712442	AB712422	USA
*Dacrymyces cerebriformis*	Dai 19,826	OM955201	OM955196	China
*Dacrymyces cerebriformis*	Dai 19832*	OM955202	OM955197	China
*Dacrymyces chiangraiensis*	MFLU 160572	KY498587	—	Thailand
*Dacrymyces chrysocomus*	UPS: F-940136	MN595629	MN595629	Spain
*Dacrymyces chrysospermus*	TUFC 13115	AB712452	AB299073	Japan
*Dacrymyces citrinus*	PDD 107915*	LC131417	LC131376	New Zealand
*Dacrymyces cylindricus*	PDD 105052*	LC131419	LC131378	New Zealand
*Dacrymyces cyrtosporus*	PDD 107980*	LC131422	LC131381	New Zealand
*Dacrymyces cyrtosporus*	PDD 107952	LC131421	LC131380	New Zealand
*Dacrymyces dictyosporus*	HHB-8618	AB712454	AB712429	USA
*Dacrymyces fennicus*	H: Miettinen 21,174	MW191957	MW159071	Finland
*Dacrymyces fennicus*	UPS: F-946596	MZ147627	MZ147627	Sweden
*Dacrymyces flabelliformis*	HHB 18308*	AB712455	AB712430	New Zealand
*Dacrymyces flavobrunneus*	AMO789*	OK257533	OK257540	Brazil
*Dacrymyces grandii*	NCSLG21158	MW191953	—	USA
*Dacrymyces grandinioides*	H7008841	MW191950	MW159076	Kenya
*Dacrymyces intermedius*	PDD 107851*	—	LC131384	New Zealand
*Dacrymyces intermedius*	PDD 107939	—	LC131385	New Zealand
*Dacrymyces invisibilis*	14597MDT*	MH230100	MH230102	Chile
*Dacrymyces invisibilis*	14617MD	MH230101	MH230103	Chile
*Dacrymyces jauensis*	FJAU68959	PP776532	PP776577	China
*Dacrymyces jauensis*	FJAU68961*	PP776534	PP776579	China
*Dacrymyces lacrymalis*	TUFC13327	AB712456	AB299069	Japan
*Dacrymyces longistipitatus*	PDD 107997*	LC131426	LC131387	New Zealand
*Dacrymyces maxidorii*	RLMA305	OK257532	OK257537	Brazil
*Dacrymyces maxidorii*	RLMA306	OK257531	OK257536	Brazil
*Dacrymyces minutus*	HNo.648	AB712459	AB472733	Japan
*Dacrymyces naematelioides*	HKAS 133174a*	PP550877	PP550024	China
*Dacrymyces naematelioides*	YAASM 7491	PP550882	—	China
*Dacrymyces novae-zelandiae*	PDD 107892	LC131427	LC131390	New Zealand
*Dacrymyces ovisporus*	H: Miettinen 20,787	MW191964	MW159074	Finland
*Dacrymyces ovisporus*	H: Spirin 11,145	MW191960	MW159073	Norway
*Dacrymyces pachysporus*	PDD 105004*	LC131429	LC131392	New Zealand
*Dacrymyces parastenosporus*	PDD 104960	LC131431	LC131394	New Zealand
*Dacrymyces parastenosporus*	PDD 104963*	LC131432	LC131395	New Zealand
*Dacrymyces pezizoides*	TNS-F-54909	LC386890	LC386894	Japan
*Dacrymyces pinacearum*	UPS: F-593533	MN595637	MN595637	Japan
*Dacrymyces puniceus*	TUFC 12833	AB712449	AB299057	Japan
*Dacrymyces pusillus*	UPS F-176774	MN595639	MN595639	Sweden
*Dacrymyces san-augustinii*	Wu544	OL587813	OL546781	China
*Dacrymyces sinostenosporus*	Dai 20003*	MW540888	MW540890	China
*Dacrymyces sinostenosporus*	Dai 20,008	MW540889	MW540891	China
*Dacrymyces sobrius*	CFMR: RLG-13487*	AB712445	AB712425	USA
*Dacrymyces spathularia*	TUFC12846	AB712473	AB472710	Japan
*Dacrymyces spathularia*	FCME 27539	MN733711	MN733722	Mexico
*Dacrymyces stenosporus*	PDD 107970	LC131434	LC131397	Japan
*Dacrymyces stenosporus*	PDD 105018*	NR_164094	NG_059171	New Zealand
*Dacrymyces stillatus*	UPS F-939814	MN595676	MN595676	Sweden
*Dacrymyces subalpinus*	TUFC 12834	AB712465	AB299060	Japan
*Dacrymyces subantarcticensis*	PDD 107988	LC131436	LC131400	New Zealand
*Dacrymyces undulatomarginatus*	HKAS 145921*	PV037615	PV037430	China
*Dacrymyces venustus*	O: Adane 150*	MW191949	MW159075	Ethiopia
*Dacryopinax elegans*	HHB-18731	AB712471	AB712433	USA
*Ditiola radicata*	UPS: F-939957	MN595641	MN595641	Sweden
*Femsjonia peziziformis*	H Haikonen 30,097	MN595642	MN595642	Finland
*Femsjonia uniseptata*	TNS-F-54019*	NR_164095	NG_060435	Japan
*Guepiniopsis buccina*	UPS F-940947	MN595643	MN595643	Spain
*Guepiniopsis estonica*	UPS: F-940137	MN595632	MN595632	Sweden

*indicates type material, holotype; — refers to the missing data; and the new species are shown in bold.

ML research analysis was conducted using RAxML-HPC v. 8.2.3 (Stamatakis, 2014) and RAxML-HPC through the CIPRES Science Gateway (Miller et al., 2009).^2^ Statistical support values (BS) were obtained using nonparametric bootstrapping with 1,000 replicates. The BI analysis was performed using MrBayes 3.2.7a (Ronquist and Huelsenbeck, 2003). A total of four Markov chains were run in two runs from random starting trees for 2 million generations until the split deviation frequency value was <0.01, and trees were sampled every 1,000 generations. The first 25% of the sampled trees were discarded as burn-in, and the remaining trees were used to reconstruct a majority-rule consensus tree and calculate Bayesian posterior probabilities (BPPs) for the clades.

The optimal substitution models for the combined dataset were determined using the Akaike information criterion (AIC) implemented in MrModeltest 2.3 (Posada and Crandall, 1998; Nylander, 2004) after scoring 24 models of evolution in PAUP* version 4.0 beta 10 (Swofford, 2002). The selected model applied for the Bayesian inference (BI) and maximum likelihood (ML) analyses was GTR + F + I + G4.

Branches with ML bootstrap support values > 70% and BPPs > 0.90 were considered strongly supported. In addition, the ML analysis produced the best tree, and only the ML tree is presented, along with the support values from the ML and BI analyses. FigTree v1.4.4 (Rambaut, 2012) was used to visualize the resulting tree.

## Results

### Phylogenetic analyses

**Figure 1:**
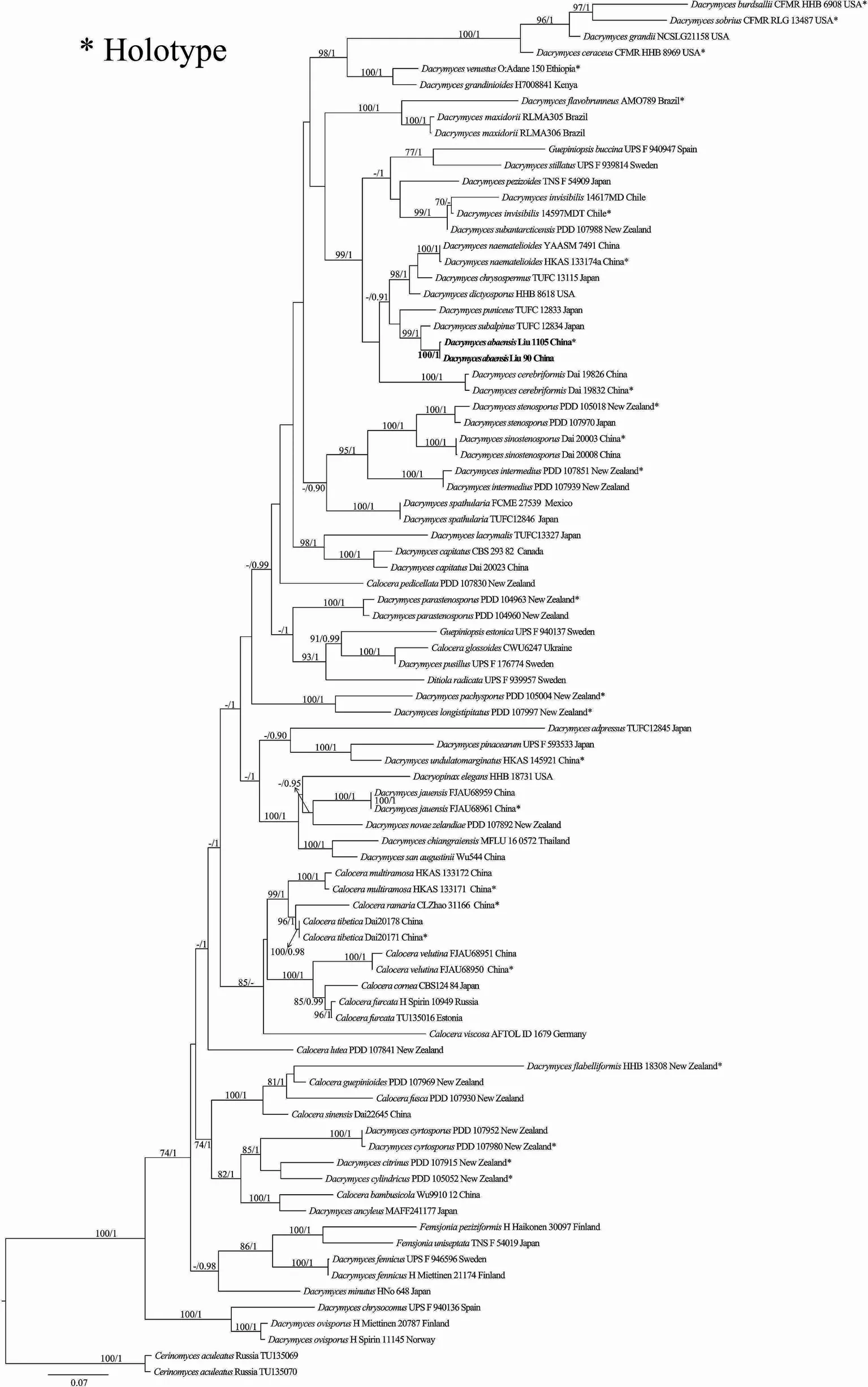
Phylogeny of *Dacrymyces* and related species generated by ML analysis based on combined ITS+nLSU sequences. Branches are labeled with ML bootstrap values > 70% and Bayesian Posterior Probabilities > 0.90, respectively. New species are shown in bold.

The concatenated ITS+nLSU dataset contained sequences from 86 fungal specimens representing 47 *Dacrymyces* taxa, and the average standard deviation of split frequencies was 0.004502 (BI). The phylogenetic tree inferred from the ITS and nLSU sequences showed that *Dacrymyces abaensis* sp. nov. clustered within the *Dacrymyces* clade and grouped with *D. subalpinus* Kobayasi, *D. naematelioides* Y. H. Ma, W. M. Chen & Y. C. Zhao, *D. puniceus* Kobayasi, *D. dictyosporus* G. W. Martin, and *D. chrysospermus* Berk. & M. A. Curtis, with moderate support (0.91 BPP; Figure 1).

### Taxonomy

**Figure 2:**
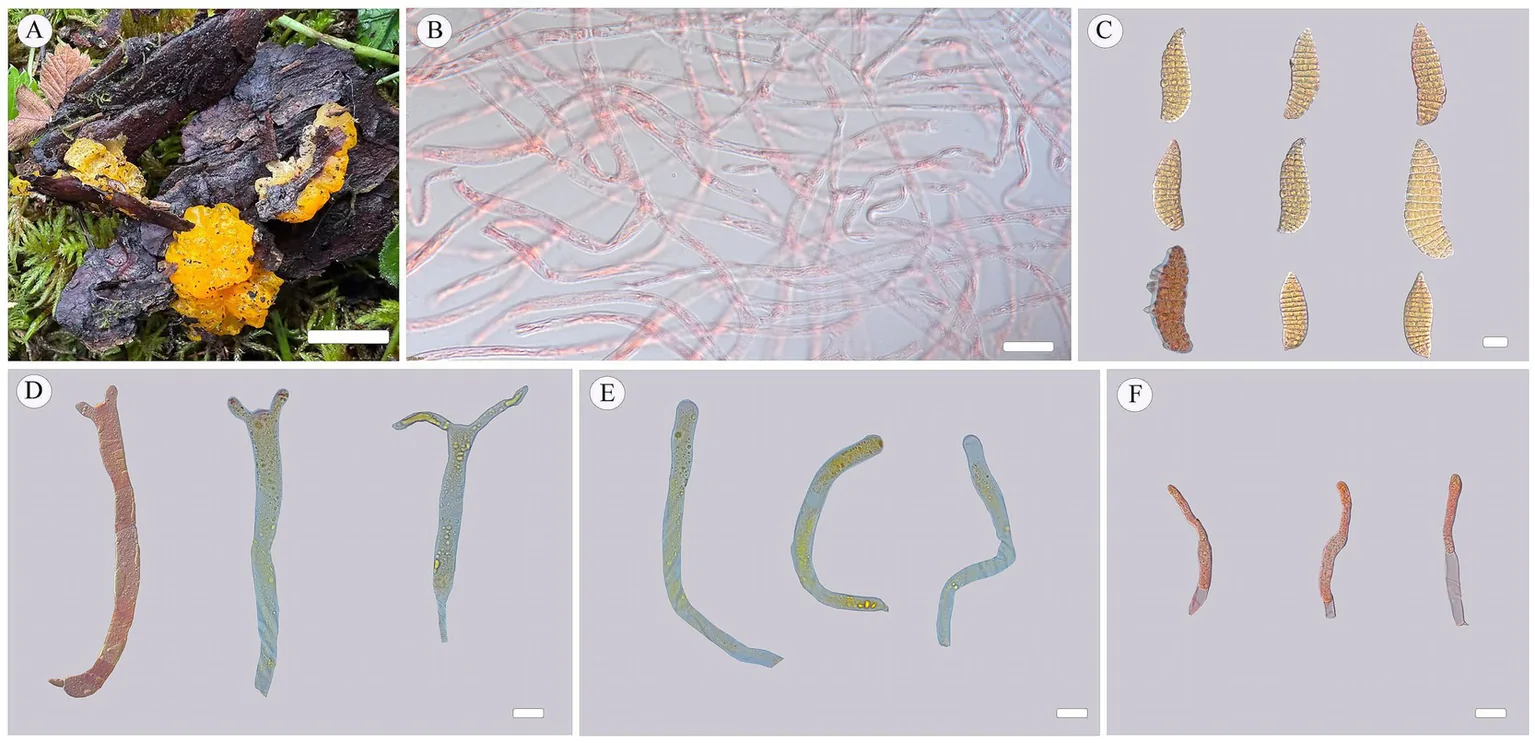
*Dacrymyces abaensis* (holotype, Liu 1105). **(A)** Basidiomata. **(B)** Internal hyphae. **(C)** Basidiospores. **(D)** Basidia. **(E)** Probasidia. **(F)** Hyphidia. Scale bars: **(A)** = 1 cm, **(B–F)** = 10 μm. Photo by Ying-Ge Fan.

*Dacrymyces abaensis* J. L. Zhang, Y. G. Fan & Z. B. Liu, sp. nov. Figure 2.

MycoBank number: MB 863632.

Type: China, Sichuan Province, Aba, Songpan County, Mounigou Scenic Area, on rotten wood of *Picea*, southwestern China, 103° 31′E, 32° 37’N, alt. 3,002 m. The vegetation is a subalpine coniferous forest. 31 Aug 2025, Z. B. Liu 1105 (holotype SAUFC 001105).

Etymology: *Abaensis* (Lat.), referring to the species being found in Aba Xizangan and Qiang Autonomous Prefecture, Sichuan Province, China.

Diagnosis: Differs from other *Dacrymyces* species by its sessile, cerebriform, orange-yellow gelatinous basidiomata; subclavate probasidia (109–120 × 5.5–9 μm) that become bifurcate at maturity; clavate, thin-walled hyphidia (39–61 × 3.5–5.5 μm); and large, navicular basidiospores (36.6–56 × 11.9–15.6 μm) with up to 15 transverse septa and several longitudinal septa.

Basidiomata: Scattered or gregarious, sometimes coalesced, sessile, cerebriform, gelatinous, and orange-yellow when fresh, becoming firm-gelatinous and apricot-orange when dry; 5 mm high and 12 mm in diameter.

Hyphal structure: Marginal hyphae not observed. Internal hyphae hyaline, thin-walled, flexuous, smooth, septate, frequently branched, interwoven, without clamp connections, and 2.5–3.5 μm in diameter.

Hymenium: Restricted to the upper surface of the basidiomata and composed of basidia, probasidia, and hyphidia. Probasidia yellow, thin-walled, subclavate, with a basal simple septum, becoming bifurcate at maturity, 109–120 × 5.5–9 μm; sterigmata up to 30 μm long; with obvious guttules. Hyphidia yellow, thin-walled, clavate, with a basal simple septum, 39–61 × 3.5–5.5 μm.

Basidiospores: Navicular, with an apiculus at the base, yellow, thin-walled, usually with up to 15 transverse septa and additional longitudinal septa at maturity, (36.1–)36.6–56(−57.3) × (11.5–)11.9–15.6(−17.3) μm, L = 44.62 μm, W = 13.43 μm, Q = 3.24–3.46 (*n* = 60/2). Germinated by the production of conidia and germ tubes.

Additional specimen examined (paratype): China, Sichuan Province, Aba, Songpan County, Mounigou Scenic Area, on rotten wood of *Picea*, southwestern China, 103° 31′E, 32° 37’N, alt. 3,002 m. The vegetation is a subalpine coniferous forest. 31 Aug 2025, Z. B. Liu 90 (SAUFC 000090).

## Discussion

Based on a comprehensive evaluation of morphological characteristics and molecular phylogeny (ITS and nLSU), we describe *Dacrymyces abaensis* as a new species. The holotype and paratype were collected from Sichuan Province, contributing to the diversity of jelly fungi in this biologically rich region of China.

Phylogenetically, *Dacrymyces abaensis* is nested within the *Dacrymyces* clade based on ITS and nLSU sequence data, and the two specimens of *D. abaensis* form a well-supported lineage (100% ML, 1.00 BPP; Figure 1). *D. abaensis* was closely related to *D. subalpinus*, *D. naematelioides*, *D. puniceus*, *D. dictyosporus,* and *D. chrysospermus* in our phylogenetic analyses, with moderate support (0.91 BPP; Figure 1), but the basidiospores of *D. abaensis* (36.6–56 × 11.9–15.6 μm) are larger than those of *D. subalpinus* (30–43 × 8–10 μm; Shirouzu and Hosoya, 2016), *D. naematelioides* (18.5–28.5 × 8.9–13.8 μm; Ma et al., 2024), *D. puniceus* (18–23 × 7–8 μm; Shirouzu and Hosoya, 2016), *D. dictyosporus* (25–28 × 10–12 μm; Martin, 1958), and *D. chrysospermus* (12–23 × 5–10 μm; Shirouzu et al., 2009). In addition, a minimum of >1.5% nucleotide differences in the ITS regions may be indicative of a new species (Jeewon and Hyde, 2016), and the nucleotide differences in the ITS regions between *D. abaensis* and *D. subalpinus* are up to 2.7%.

Regarding the distribution of *Dacrymyces* in China, *D. sichuanensis* B. Liu & L. Fan is currently the only other species recorded in Sichuan Province, aside from our new species. However, *D. abaensis* is morphologically distinct from *D. sichuanensis*. The latter is characterized by smaller, 0–3-septate basidiospores measuring 12.5–15.6 × 4.5–6.5 μm (Liu and Fan, 1990). In contrast, the basidiospores of *D. abaensis* are significantly larger (36.6–56 × 11.9–15.6 μm) and develop up to 15 septa at maturity. These distinct morphological differences, particularly in spore dimensions and septation, clearly separate our new species from *D. sichuanensis*.

## Data Availability

The datasets presented in this study can be found in online repositories. The names of the repository/repositories and accession number(s) can be found in the article/supplementary material.

## Footnotes

1
http://mafft.cbrc.jp/alignment/server/

2
http://www.phylo.org
